# Enhanced Antifouling in Flat-Sheet Polyphenylsulfone Membranes Incorporating Graphene Oxide–Tungsten Oxide for Ultrafiltration Applications

**DOI:** 10.3390/membranes13030269

**Published:** 2023-02-24

**Authors:** Raghad M. Al-Maliki, Qusay F. Alsalhy, Sama Al-Jubouri, Adnan A. AbdulRazak, Mohammed Ahmed Shehab, Zoltán Németh, Klara Hernadi, Hasan Sh. Majdi

**Affiliations:** 1 Membrane Technology Research Unit, Department of Chemical Engineering, University of Technology-Iraq, Alsinaa Street 52, Baghdad 10066, Iraq; 2 Department of Chemical Engineering, College of Engineering, University of Baghdad, Aljadria, Baghdad 10071, Iraq; 3 Faculty of Materials and Chemical Engineering, University of Miskolc, H-3515 Miskolc, Hungary; 4 Polymers and Petrochemicals Engineering Department, Basrah University for Oil and Gas, Basrah 61004, Iraq; 5 Advanced Materials and Intelligent Technologies Higher Education and Industrial Cooperation Centre, University of Miskolc, H-3515 Miskolc, Hungary; 6 Institute of Physical Metallurgy, Metal Forming and Nanotechnology, University of Miskolc, H-3515 Miskolc, Hungary; 7 Department of Chemical Engineering and Petroleum Industries, Al-Mustaqbal University College, Babylon 51001, Iraq

**Keywords:** polyphenylsulfone, polyvinylpyrrolidone, graphene oxide, tungsten oxide, antifouling, BSA removal, mixed matrix membrane, ultrafiltration membrane

## Abstract

In this study tungsten oxide and graphene oxide (GO-WO_2.89_) were successfully combined using the ultra-sonication method and embedded with polyphenylsulfone (PPSU) to prepare novel low-fouling membranes for ultrafiltration applications. The properties of the modified membranes and performance were investigated using Fourier-transform infrared spectroscopy (FT-IR), scanning electron microscopy (SEM), contact angle (CA), water permeation flux, and bovine serum albumin (BSA) rejection. It was found that the modified PPSU membrane fabricated from 0.1 wt.% of GO-WO_2.89_ possessed the best characteristics, with a 40.82° contact angle and 92.94% porosity. The permeation flux of the best membrane was the highest. The pure water permeation flux of the best membrane showcased 636.01 L·m^−2^·h^−1^ with 82.86% BSA rejection. Moreover, the membranes (MR-2 and MR-P2) manifested a higher flux recovery ratio (FRR %) of 92.66 and 87.06%, respectively, and were less prone to BSA solution fouling. The antibacterial performance of the GO-WO_2.89_ composite was very positive with three different concentrations, observed via the bacteria count method. These results significantly overtake those observed by neat PPSU membranes and offer a promising potential of GO-WO_2.89_ on activity membrane performance.

## 1. Introduction

Treatment of wastewater generated by various sectors, such as petrochemicals, metallurgy, food, pharmaceuticals, and other pollutants, has become a major problem across the world. Wastewater discharged without treatment has a variety of negative consequences on the environment, damaging surface water, subsurface water, and soil. Furthermore, due to the evaporation of hydrocarbons, there is a risk of air pollution. Direct discharge of wastewater into the environment is equally harmful to the ecosystem [1,2,3,4]. 

Among wastewater treatment technologies, membrane filtration is one of the most promising methods, which provides a great variety of tunable parameters to ensure customized application [5]. Membranes are made by combining cellulose acetate [6], acrylonitrile butadiene styrene [7], and polyvinyl alcohol with adsorptive polymers, such as chitosan and polypyrrole, to increase their adsorptive qualities [8], polyethersulfone (PES) [9,10,11], polyvinylidene fluoride (PVDF) [12,13], polyphenylsulfone (PPSU), and polyvinyl chloride (PVC) [14,15,16]. In addition to that, it should be highlighted that nanoparticles have been frequently used in the construction of nanocomposite membranes to improve their performance [17,18]. The addition of nanoparticles into polymeric membranes has many positive effects on the unique properties of the membrane because these particles have special features, such as a large surface area, permeability and selectivity, and hydrophilic properties [19,20,21]. Titanium dioxide (TiO_2_) [22], silica (SiO_2_) [23,24], iron oxides [25], zinc oxide (ZnO) [26], activated carbon [27], carbon nanotube (CNT) [28], zeolite [19,29,30,31], MWCNTs incorporating with GO [32], and graphene oxide (GO) [33] are often combined with polymers to make a special membrane that is used for a special separation, such as oil separation [1,2] or dye separation [3,4,34].

Polymers belonging to the polysulfone family (in particular, polysulfone (PSF) [35], polyethersulfone (PES) [36], and polyphenylsulfone (PPSU) [37]) have gained importance in commercial membrane technology over the last two decades due to their exceptional properties, including thermal, hydrolytic and mechanical stability, chemical resistance, and film-forming ability. Furthermore, the ability to bulk modify the polymer skeleton as well as customize membrane porosity and pore size make polysulfone polymers suitable for a wide range of filtering applications, from ultrafiltration [38] to reverse osmosis (RO) [39]. Because of its unique advantages, such as chemical stability, solvent resistance, improved mechanical properties, hydrophobicity, and high-temperature resistance, polyphenylsulfone (PPSU) [40] was selected as the best polymeric membrane material [37,41].

Chemical corrosion resistance and mechanical strength are two more benefits of PPSU. However, it has low surface energy, which limits the antifouling ability and reduces permeate flow and selectivity, resulting in higher operating expenses. Membrane fouling can be classified into two types: reversible and irreversible fouling. The low interaction of foulants on the membrane surface causes reversible fouling, which may be removed by simple washing, while irreversible fouling is produced by heavily clogged membrane pores that cannot be cleaned then, reducing the membrane’s operational life and lowering the separation performance. For practical applications, improving the hydrophilicity and antifouling ability of the PPSU membrane has been a research focus in recent years by incorporating an inorganic nanoparticle additive into the organic membrane material to create a mixed matrix membrane [42,43].

One of the most promising materials is GO and WO_x_ contains several functional groups, including epoxides and hydroxyls (the most hydrophilic of all atomic sheets), which ensures excellent compatibility with a wide range of polymers. However, GO is naturally hydrophilic, and embedding it in polymeric membranes would reduce wettability and increase antifouling [2]. In the same regard, the membrane has a solid atom-thin structure, with functional groups of oxygen and interlayer spacing, so introducing GO into the polymer network can accelerate water movement through the membrane and enhance the membrane’s resistance against high operating pressures [32].

Different functional groups, such as carboxyl, epoxy, and hydroxyl, can be placed at the edges and basal planes of graphene-based nanomaterials to easily synthesize them. These nanomaterials’ two unique qualities have drawn attention to the creation of nanocomposite membranes. To begin with, the majority of GO derivatives have high charge densities, which help achieve stable distribution in organic solutions, such as DMAc solution. In addition, because of their various oxidation states, they have tunable hydrophilic properties that may be added to polymer materials to create high-performance membranes with flux and antifouling properties to meet the needs of particular water treatment applications [44]. There have been earlier studies on the development of the impact of GO nanofillers on membrane characteristics. Ganesh et al. [45] found an increase in salt removal as well as pure water flux by adding GO to a PSF polymeric membrane, Yu et al. [46] enhanced the tensile strength as well as antifouling characteristics with less permeation by adding HPEI-GO to PES, Lee et al. [47] improved the antifouling qualities of the advanced membrane bioreactor (MBR), adding GO to the PSF polymeric membrane, and Zhao et al. [48] added GO to a PVC membrane to improve the mechanical properties, water flux, and hydrophilic nature of the membrane. Wu et al. [49] improved permeation, protein rejection, and antifouling capability by incorporating GO with SiO_2_ and adding them to the PSF polymeric membrane. Zinadini et al. [50] enhanced the properties for water flux, dye rejection, and antibiofouling by adding GO to a PES polymeric membrane. As can be seen, the inclusion of GO nanoparticles has generally enhanced the mechanical, permeability, as well as antifouling characteristics of polymer membranes. However, the use of a GO nanostructure with specific geometric shapes and chemical properties was the primary factor in these results, and it is still unknown how the shape as well as the oxidation state of the nanofillers will affect the membrane properties.

Tungsten oxide (WO_x_) x ≤ 3 nanoparticles have attracted a lot of attention, because of their abundance, strong oxidation capabilities, as well as chemical stability at appropriate pH values. WO_3_ is also known for its nonstoichiometric characteristics, as the lattice can hold a number of oxygen vacancies (WO_2_, WO_2.72_, WO_2.8_, WO_2.9_, and WO_3_). In many sectors, including heat generation, photocatalysis, as well as energy-related and gas sensor applications, tungsten-oxide-based materials and their hybrids also garnered a lot of interest. Saha et al. [51] studied the performance of proton exchange membrane fuel cells by W_18_O_49_ NWs grown on carbon paper, and then Pt precursors were reduced with glacial acetic acid to create composite electrodes of Pt nanoparticles. In comparison to a conventional Pt/C electrode, the Pt/W_18_O_49_ NW/carbon paper composite electrode shows high kinetic activity for oxygen reduction reaction in a 1 cm^2^ single cell and higher CO tolerance. It may be possible to fabricate new electrodes with enhanced quality, lower cost, and higher CO acceptance for PEMFCs and direct methanol membrane fuel cells by extending such nanowire-based 3D electrodes to other material classes. Abdullah et al. found the inclusion of hydrophilic WO_2.89_ nanomaterial [52] limited membrane fouling throughout the photocatalytic process, and reactive radicals could react with adsorbate species and reject them, increasing the membrane’s hydrophilic nature [53]. Sathya et al. [54] also found that adding WO_3_ greatly improved the biofouling properties of a PEI membrane by using BSA and humic acid as a model sample of pollutants in wastewater. 

Fouling can induce membrane surface degradation, resulting in a decrease in flow. By integrating metal oxides with base membranes, fouling may be decreased, and surface connectivity can be increased. Tungsten oxide is a well-known photocatalyst with anti-bacterial properties, which can lead to reduced biofouling [54]. Graphene oxide has high hydrophilic properties so it will help in reducing fouling. In addition, the antibacterial property of WO_2.89_ helps to compensate for the shortcomings of GO due to its antibacterial weakness, and, on the other hand, the incredible antifouling property of GO helps the PPSU membrane to be both antifouling and antibacterial, which can, thus, be used in the future to treat biological contamination. The antifouling property of the PPSU membrane with the addition of GO-WO_2.89_ as nanoparticle additives was also studied in the current work. In this work, a (GO-WO_2.89_) nanocomposite was successfully synthesized for the first time and embedded in the PPSU casting solution to improve the structural morphology and performance of PPSU membranes for ultrafiltration applications. 

## 2. Materials and Methods

### 2.1. Materials

Polyphenylsulfone, Ultrason^®^ P (PPSU), with an average molecular weight of 48,000 and transition temperature Tg = 220 °C, was supplied by BASF, Ludwigshafen, Germany. Polyvinylpyrrolidone (PVP) polymer (MW = 40,000 g/mol) was obtained from Kemphasol Co. and Alpha Chemic (Mumbai, India). Dimethylactamide (DMAC) was used as a solvent. Tungsten oxide WO_2.89_ (W_19_ O_55_), with an average particle size of 80−100 nm and purity of 99.9%, was obtained from Hongwu International Group Ltd. (Guangzhou, China). Bovine serum albumin (BSA) was received from Avonchem (Macclesfield, UK).

### 2.2. Preparation of Nanoparticle Composite (GO-WO_2.89_)

**Figure 1:**
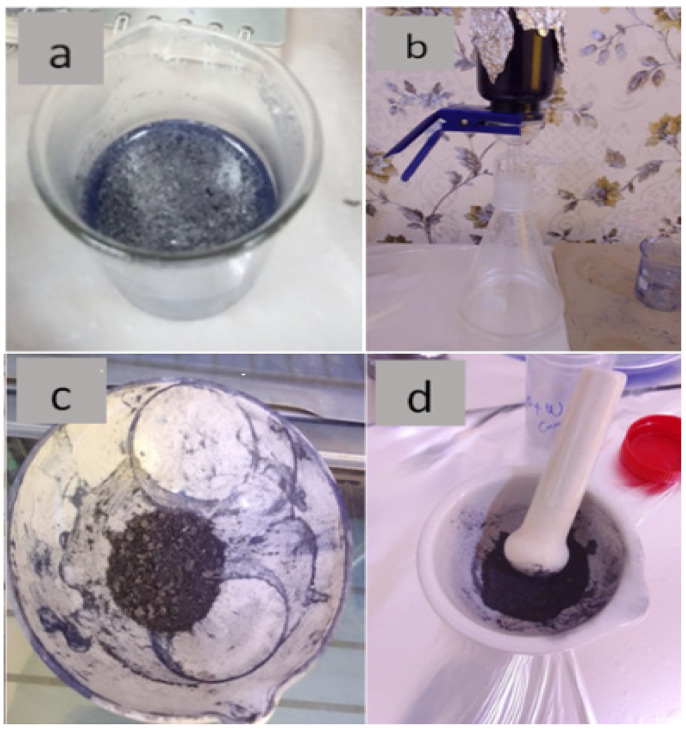
(**a**) GO and WO_2.89_ after mixing, (**b**) vacuum nanofiltration sample, (**c**) sample after drying, (**d**) sample after grinding.

**Figure 2:**
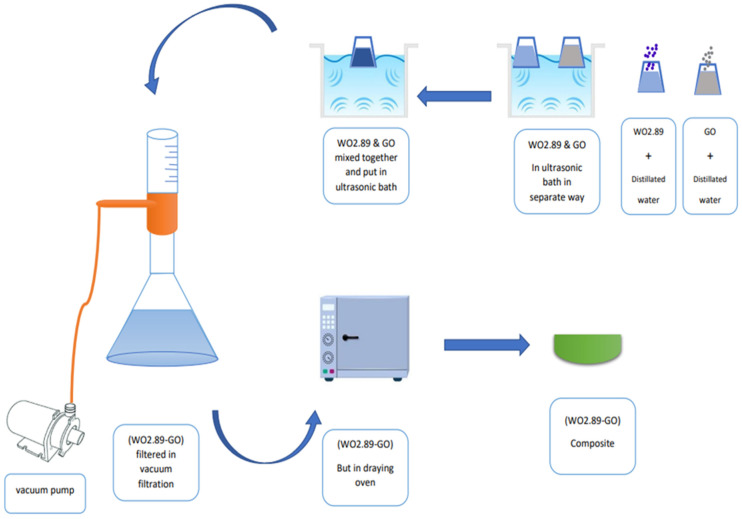
Scheme of manufacturing process of nanocomposite (WO_2.89_-GO).

Graphene oxide (GO) was prepared by modified Hummer’s method, as previously reported [32]. WO_2.89_-GO nanocomposite was successfully synthesized using the ultrasonication method, as shown in Figure 1. The preparation scheme shown in Figure 2 was used to fabricate the samples, following the procedure described by Jeevitha et al. [55]. Briefly, two solutions were made by dispersing 70 mg of WO_2.89_ in 70 mL of distilled water and 210 mg of GO in 210 mL of distilled water, and then mixed and transferred to an ultrasonication bath for 2 h. The products were collected by vacuum filtration and drying at 100 C for 1 h.

### 2.3. Preparation of Flat-Sheet Membrane

The phase inversion method was used to prepare the mixed matrix membranes. Two types of mixed matrix membrane were prepared. For the first one, 17% PPSU was used, and for the second one, 15% PPSU, and 2% PVP was used to obtain the total polymer 17%. The different weight percentage range of GO-WO_2.89_ powder was used throughout the membrane manufacturing operation, such as (0, 0.5, 1, 1.5, 2) wt.%. The compositions of the nanocomposite membranes are shown in Table 1.

The polymers PVP and PPSU were dried for 1 h at 75 °C, introduced to DMAc solvent, and then stirred until completely dissolved at room temperature. GO-WO_2.89_ powder was dispersed into the solution with continuous stirring for 1 h at room temperature. Then, the casting solution was put in an ultrasonic device for 30 min to degas the bubbles that could form in the casting solution and to make a high dispersion for the material. After that, the casting solution was placed on a clean glass plate using a casting knife (AFA IV, Shanghai, China) set to a 200 µm air gap. The resultant film was rapidly soaked in a distilled water coagulating bath at 25 °C to remove the solvent and harden the resultant thin-film membrane. Then, the membrane was kept in a deionized water container.

### 2.4. UF Membranes and Systems

**Figure 3:**
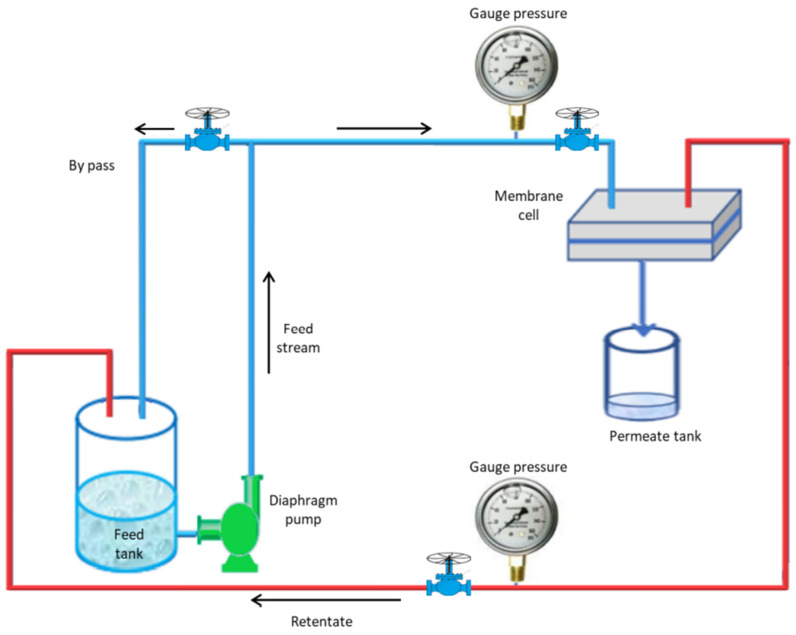
Schematic diagram of the experimental nanofiltration system.

The experimental setup contains the following main parts: the membrane cell with a 2 mm channel gap, gauge pressure, diaphragm pump, valves, feed tank for the pure water and feed tank for BSA solution, and permeate tank, with 2 mm channel gap. The performance of the prepared composite membrane was tested using a cross-flow UF membrane unit, as shown in Figure 3. Membrane cell samples had an active area of 14.4 cm^2^. In the beginning, all membranes were operated for 15 min at 4 bar using pure distilled water with 1.2 L/min cross-flow velocity. The pressure was dropped to 2 bar to achieve a steady state, and water flow was measured for each membrane to evaluate the pure water flux (PWF). The BSA solution was then passed through the membranes at 2 bar and the flux was evaluated every 15 min. The prepared membranes were cut to the desired size for flux and rejection tests, put inside the membrane module, and compressed before taking the measurements. Using the filtration system depicted in Figure 3, the clean water flux and the BSA solution of the modified membranes and the control PPSU were measured. 

Equations (1) and (2) [56] were used to calculate the permeate flow and rejection % of all membranes.

(1) $$J=\frac{V}{A*t}$$

where *V* represents the permeate flow rate (L), *t* represents the time of filtration (s), and *A* represents the effective membrane’s surface area (m^2^). The tank was discharged and recharged with the BSA solution after the pure water experiment. The membranes’ performance was studied in flux and rejection. To investigate the impact of experimental parameters on BSA removal, the filtration process was carried out under the following conditions: temperature of 25 °C, feed solution concentration of 1 g/L, and a cross-flow UF system at lab scale. After reaching a steady-state pressure of 2 bar, the separation process began and continued for 1 h.

(2) $$R\left(\%\right)=\left(1-\frac{C_{P}}{C_{F}}\right)*100$$

*R* (%) is the BSA rejection percentage, and *C_p_* and *C_f_* indicate the specific activity of the permeate and feed solution, respectively.

After each testing cycle, the membrane was put in distilled water for 2 h to remove the residue BSA solution before the fouling test, and the unit was washed with distilled water for approximately 30 min to remove the leftover BSA; a clean-up operation was carried out using pure distilled water to test the fouling for each membrane and how much BSA solution was affected on the flux of the membrane. We evaluated the clean water flow every 15 min and compared it to the first clean water flow we tested earlier. 

### 2.5. Characterization of Membranes

The structural morphology of the synthesized samples was examined the QUANTA INSPECT F50 scanning electron microscope (Inspect f 50-FEI Company, Eindhoven, The Netherlands). Liquid nitrogen was employed during membrane sampling to generate clear membrane cross sections. FT-IR, a chemical identification technique, was also used (IR—BRUKER Company, Karlsruhe, German).

The water contact angle of the samples was calculated using optical equipment (C.N., Si-plasma CAM 110, Taiwan Equipment Company, Tainan, Taiwan). The porosity of the membranes was estimated from Equation (3) [11]:

(3) $$\varepsilon \left(\%\right)=\left(1-\frac{\rho_{m}}{\rho_{p}}\right)$$

*ρ_p_* and *ρ_m_*, (g.cm^−3^) are the polymer blend density and the density of the membrane, respectively.

After washing the membrane with distilled water for 30 min, the filtration was resumed. The pure water flux measurement was repeated for 1 h at a pressure of 2 bar. Equation (4) [57] was used to calculate the flux recovery ratio (*FRR*) of the membrane.

(4) $$FRR\;\left(\%\right)=\frac{J_{w1}}{J_{w2}}\times 100$$

where *J*_*w*1_ (g/m^2^·s) is the initial pure water flux and *J*_*w*2_ (g/m^2^·s) pure water flux after washing

(5) $$R_{t}=R_{r}+R_{ir}$$

(6) $$R_{r}=\left(\frac{J_{w2}-J_{p}}{J_{w1}}\right)\times 100$$

(7) $$R_{ir}=\left(\frac{J_{w1}-J_{w2}}{J_{w1}}\right)\times 100$$

The fouling and concentration polarization effects are represented by the *R_t_*. Because they are difficult to distinguish, these two effects are regarded as a single factor. The fouling resistance (*R_t_*) is assumed to be the sum of the reversible (*R_r_*) and irreversible (*R_ir_*). (*J_p_*) represent the BSA solution permeate flux (g/m^2^·s) (Equations (5)–(7)) [57,58].

## 3. Results and Discussion

**Figure 4:**
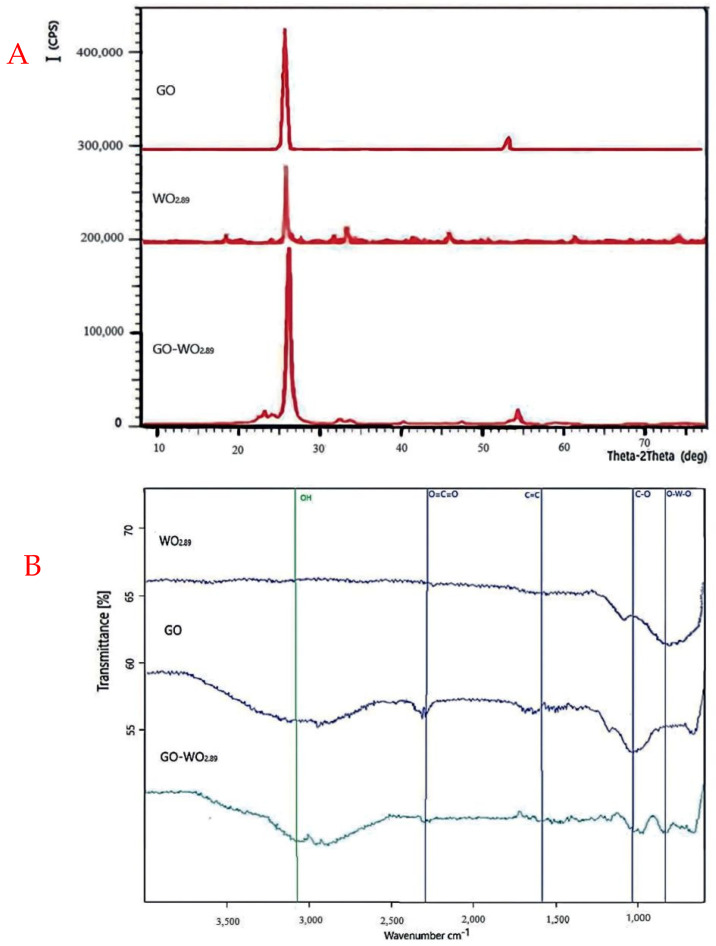
(**A**) XRD analysis of (GO, WO_2.89,_ and GO−WO_2.89_), (**B**) FT−IR analysis of nanoparticles.

XRD was performed to investigate the crystal structure and identify the material. Figure 4A shows that GO has two diffraction peaks, the highest one at 26 and the lowest one at 55. However, the tungsten oxide showed diffraction peaks at 20, 26, 35, 45, and 60, which confirm the development of the monoclinic phase of WO_2.89_ nanoparticles. Most of the prominent peaks are sharp, indicating high crystallinity in the WO_2.89_ nanoparticles.

The XRD pattern of the GO-WO_2.89_ composite shows the presence of both WO_2.89_ and GO compared with XRD patterns of pure WO_2.89_ and GO-WO_2.89,_ with no significant differences. This suggests that the phase purity of WO_2.89_ is preserved after composite synthesis with GO, which is favorable for photocatalytic activity. Because carbon has a weak crystalline structure, a low-intensity peak at 26 indicates the formation of GO sheets. The additional peaks confirmed the presence of WO_2.89_ particles at 2θ of 23, 34.1, 41.2, and 55. GO-WO_2.89_ nanocomposite XRD pattern shows that WO_2.89_ may be spread evenly on GO sheets or integrated between the sheets.

Figure 4B shows that the FT-IR spectra of the GO-WO_2.89_ composite exhibit a slight reduction in the peaks of O–H and C=O (Carbon group) stretching vibration bands in comparison to pure GO sheets. This might be owing to the presence of WO_2.89_ nanoparticles on the graphene layers. Peaks at 1062 cm^−1^ and 1086.53 cm^−1^ are related to the C–O stretching vibration band due to the presence of graphene oxide layers through the composition. The band at 1627.33 cm^−1^ is related to the unsaturated structure of C=C. The broad absorption peaks at <1000 cm^−1^ indicate the presence of pure WO_2.89_. The peaks observed at 711, 867.23, and 899 cm^−1^ are due to O–W–O stretching vibrations. The strong band at 2340 cm^−1^ is related to O=C=O (carbon dioxide) for GO, but this band cannot be observed at WO_2.89_ at the same time; this band appears weak at GO-WO_2.89_, confirming that GO and WO_2.89_ bind together. Jeevitha et al. [55] reported that tungsten oxide nanoparticles bound with the GO nanostructure, and its oxygen-containing functional groups act as a support to adsorb materials on its surface. 

**Figure 5:**
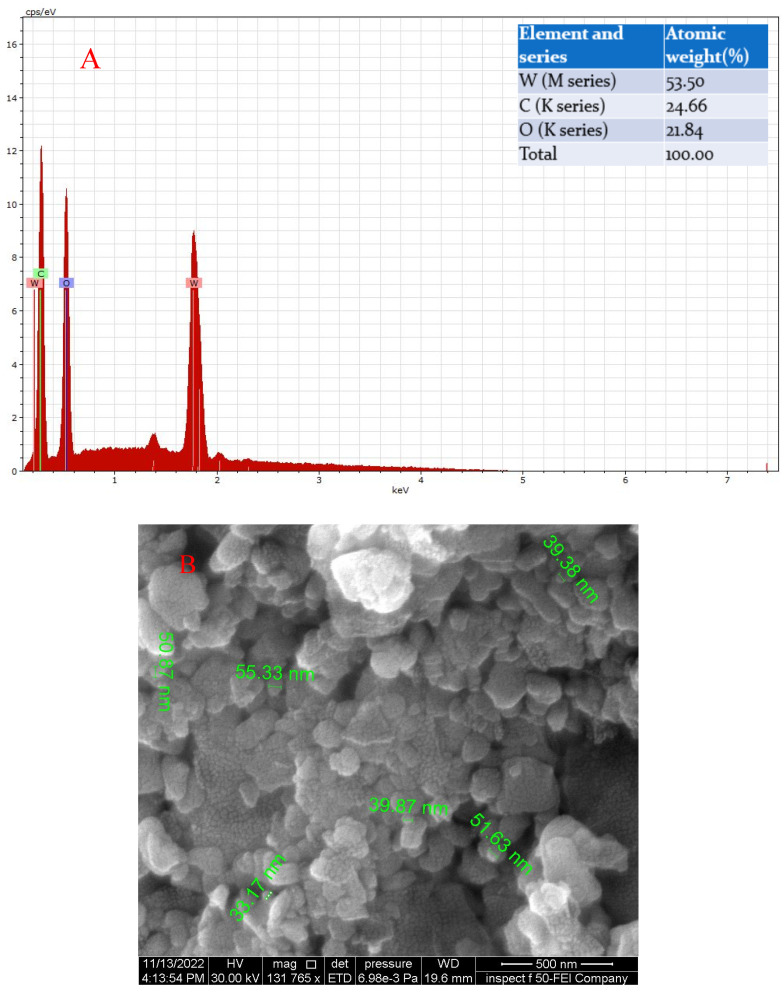
(**A**) The EDX of GO-WO_2.89_ nanoparticles and (**B**) the SEM analysis of composite GO-WO_2.89._

Figure 5B shows an FESEM image of GO-WO_2.89_ nanoparticles used to determine the average particle size of the GO-WO_2.89_ nanoparticles, which was 54 nm, demonstrating that WO_2.89_ is tightly enveloped within the layer of GO in addition to being attached to the surface of the GO sheet. The performance of FESEM is improved by the presence of multiple connections between WO_2.89_ and the GO sheet. Geng et al. [59] improved the GO sheets that are wrapping the WO_3_ particles. Because of the significant increase in the specific surface area caused by the nanoparticles in SPPS coatings and the gauze-like sheet structure of GO, adsorption and active site enhancement are greatly improved. Jeevitha et al. [55] found that the morphology of the WO_3_ nanospheres is slightly altered by ultrasonication as they are attached to the layers of graphene sheets. Van der Waals forces are used to hold the WO_3_ nanospheres to the graphene sheets. The surface area of WO_3_ is smaller when compared to the GO due to its small surface area and particle size of 150 nm, although a greater surface area greatly aids in improving a material’s performance. Theoretically, graphene has a very high surface area (2600 m^2^ g^−1^), making it an advanced alternative material for photocatalytic as well as antibacterial and anti-cancer applications.

The EDAX analysis of the GO-WO_2.89_ composite shows the presence of peaks corresponding to W, C, and O atoms. The atomic weight % of the sample is shown in Figure 5A. 

**Figure 6:**
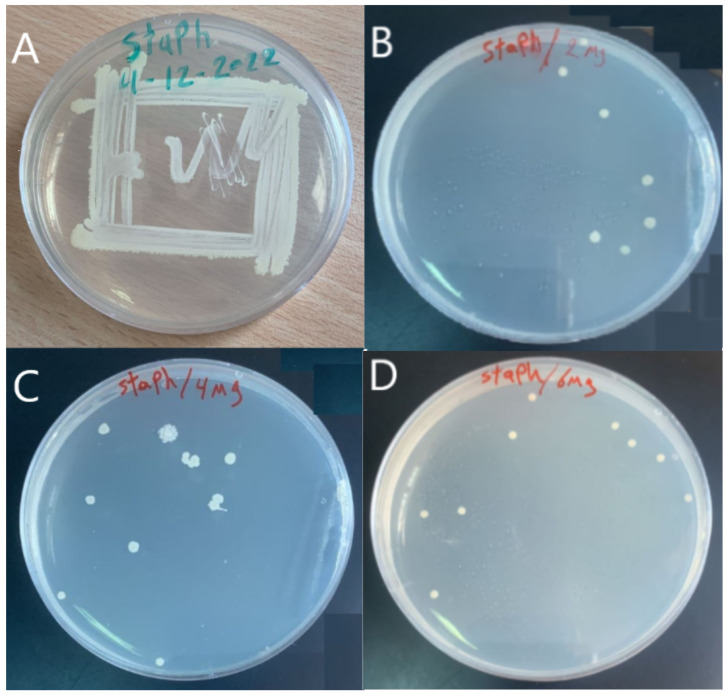
(**A**) Staphylococcus aureus (Gram-positive), (**B**) effect of 2 mg (GO-WO_2.89_), (**C**) effect of 4 mg (GO-WO_2.89_), (**D**) effect of 6 mg (GO-WO_2.89_) on Staphylococcus aureus (Gram-positive) bacteria.

The antibacterial activity of the membrane was tested using Staphylococcus aureus (Gram-positive) bacteria in Figure 6A. Figure 6B–D show the effect of the GO-WO_2.89_ composite on the bacteria in 2 mg, 4 mg, and 6 mg of GO-WO_2.89,_ respectively. It can be noticed that the antibacterial performance of the GO-WO_2.89_ composite is very positive, with three different concentrations by the bacteria count method, as shown in Table 2. The percent of the killing of bacteria was calculated according to Equation (8), where ($10^{7})$ is the percentage of dilution before being incubated, obtained by taking three dilutions ($10^{3}$) of the substance after incubating for 24 h in the vibrating incubator. Thus, 100 µm of the incubated bacteria was also placed in a culture dish. After 24 h of vibrating incubation with three dilutions of the nanoparticle, bacteria were collected and put in a culture plate. The plate was observed after an incubation time of 36 C. A distinct zone of inhibition served as a marker for the antibacterial activity and provided an approximate value. As shown in Figure 6, the zone of inhibition test was used in an antibacterial study for the prepared nanoparticles, and the clear zone indicates the nanoparticle’s antibacterial properties. It is important to mention that GO was used to have a better interface for dispersion to obtain membranes with improved hydrophilic nature and fouling control that could be used in a variety of purification and separation application fields [60]. Equation (8) was used to calculate the percentage of killing bactericidal efficacy.

(8) $$\mathrm{KOB}\;\left(\%\right)=100\%-\frac{\mathrm{NOB}\;\times 10^{3}\times 10^{2}\times 100\%}{10^{7}}$$

where KOB represents the percent of bacterial killing by the nanoparticles, and NOB represents the remaining bacteria after killing. 

**Figure 7:**
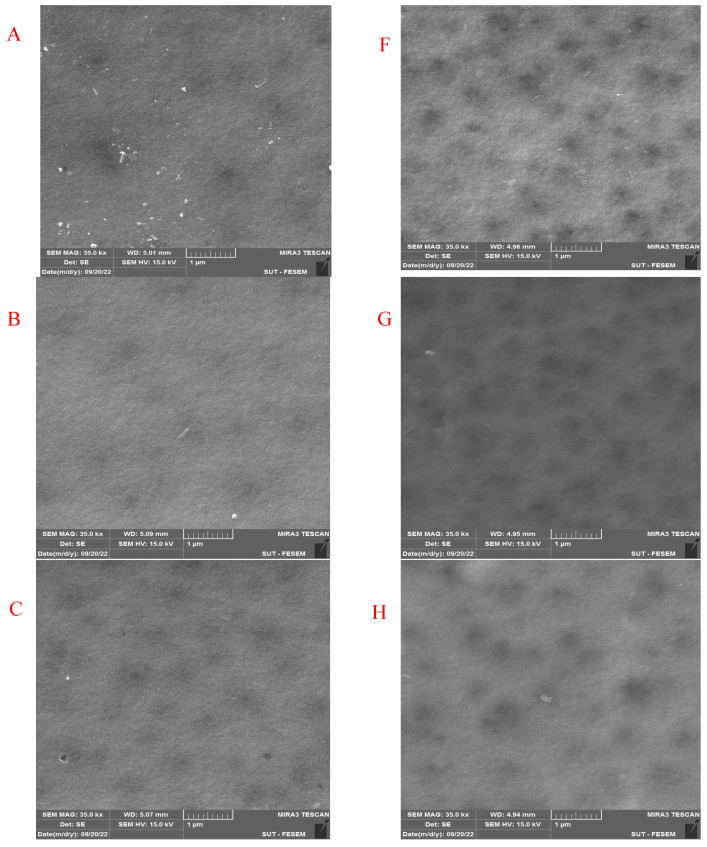

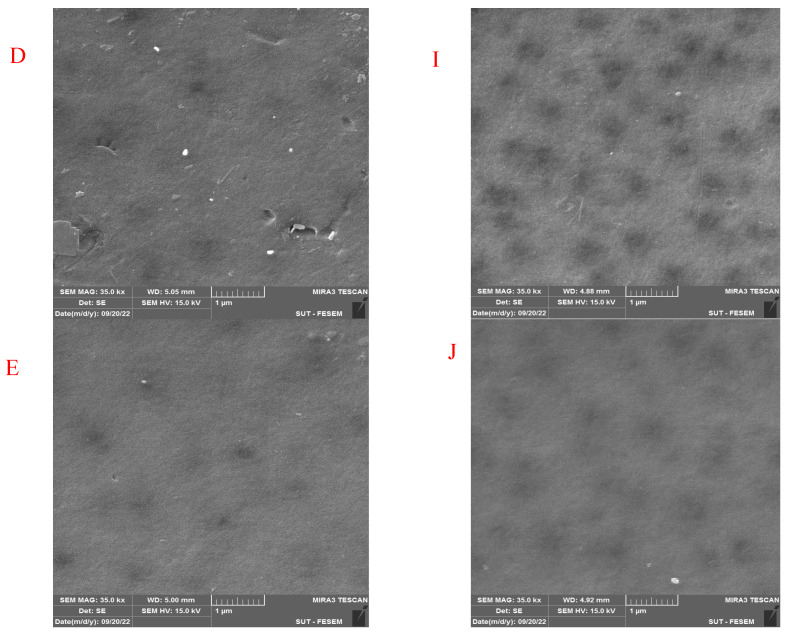
FESEM surface images for (**A**) pure PPSU membrane, (**B**) with 0.05% GO-WO_2.89_/PPSU (**C**) with 0.1% GO-WO_2.89_/PPSU, (**D**) with 0.15% GO-WO_2.89_/PPSU, (**E**) with 0.2% GO-WO_2.89_/PPSU, (**F**) pure PPSU/PVP membrane, (**G**) with 0.05% GO-WO_2.89_/PPSU/PVP (**H**) with 0.1% GO-WO_2.89_/PPSU/PVP, (**I**) with 0.15% GO-WO_2.89_/PPSU/PVP, (**J**) with 0.2% GO-WO_2.89_/PPSU/PVP.

**Figure 8:**
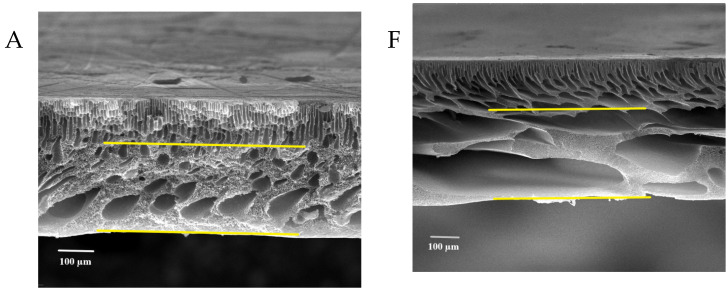

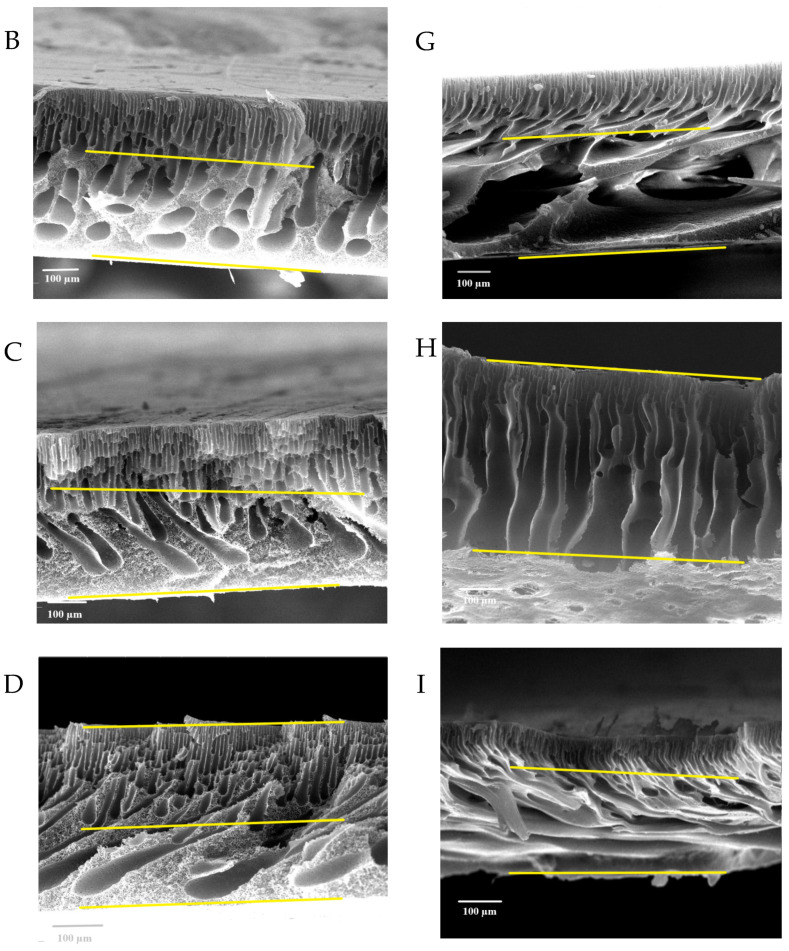

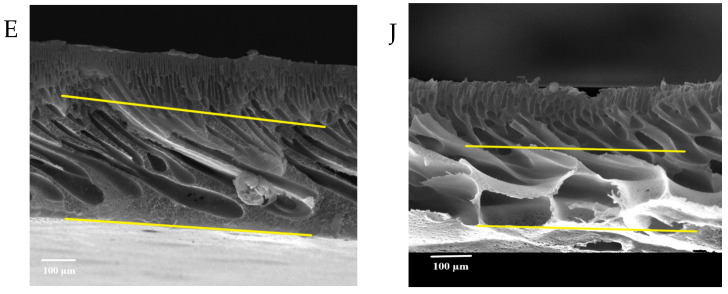
FESEM cross-sections for (**A**) pure PPSU membrane, (**B**) with 0.05% GO-WO_2.89_/PPSU, (**C**) with 0.1% GO-WO_2.89_/PPSU, (**D**) with 0.15% GO-WO_2.89_/PPSU, (**E**) with 0.2% GO-WO_2.89_/PPSU (**F**) PPSU/PVP membrane, (**G**) with 0.05% GO-WO_2.89_/PPSU/PVP, (**H**) with 0.1% GO-WO_2.89_/PPSU/PVP, (**I**) with 0.15% GO-WO_2.89_/PPSU/PVP, (**J**) with 0.2% GO-WO_2.89_/PPSU/PVP.

Figure 7 shows the FESEM images of the pure and modified membrane surfaces with varying GO-WO_2.89_ contents for the PPSU and PPSU/PVP membranes, respectively. GO-WO_2.89_ nanoparticles increase the pores for the PPSU membrane when increasing the additional amount of nanoparticles, as it can be noticed that, when added PVP to the PPSU membrane, they increase the number of pores compared with the PPSU membrane without PVP polymer because the PVP polymer is considered as pore forming. The membrane’s thickness is an important factor that influences permeability overall membrane separation techniques since it acts as a barrier to compound the transfer across the membrane wall. To improve membrane performance in ultrafiltration applications, the membrane should be appropriately thin when taking mechanical characteristics into account [4]. Figure 8A–E show FESEM images of pure PPSU and the nanocomposite membrane cross-section with varying GO-WO_2.89_ loading. As can be seen in the cross-section in Figure 8A, finger-like pores formed near the top surface, whereas a sponge layer formed near the bottom layer of a cross-section on the neat PPSU-prepared membrane. The size of finger-like pores is smaller in Figure 8A,B compared with Figure 8C. It can be noticed that the pores in the sponge layer are like tear pores in the pure PPSU membrane and started to be a finger-like structure, as seen in Figure 8C, where the microvoids increased and the sponge-like structure started to decrease with the addition of 0.05 wt. % GO-WO_2.89_ nanoparticles. When the concentration of GO-WO_2.89_ nanoparticles in the casting solution increased to 0.1 wt.%, increasing the amount of GO-WO_2.89_ nanoparticles in the casting solution to 0.15 and 0.2 wt.%, the transformation of the sponge-like structure of the PPSU increased, as shown in Figure 8D,E. Similarly, Figure 8F–J illustrate that the finger-like structure of PPSU/PVP became wider and more porous due to the pore-forming PVP additives. An increase in the amount of GO-WO_2.89_ nanoparticles increased the finger-like structure and the pores in the sponge layer decreased and became a thin finger-like structure at (0.05 and 0.1) wt.%. This is because the presence of GO-WO_2.89_ nanoparticles in the polymer solution caused a delay in the liquid–liquid solvent exchange process between the polymer solution and a (water) non-solvent. Yang et al. [61] maintained that at low concentrations of nanoparticles, microvoids expanded and become permeable, but they decreased or disappeared at high concentrations of nanoparticles. The thickness and the structure porosity of each membrane are shown in Table 3. 

**Figure 9:**
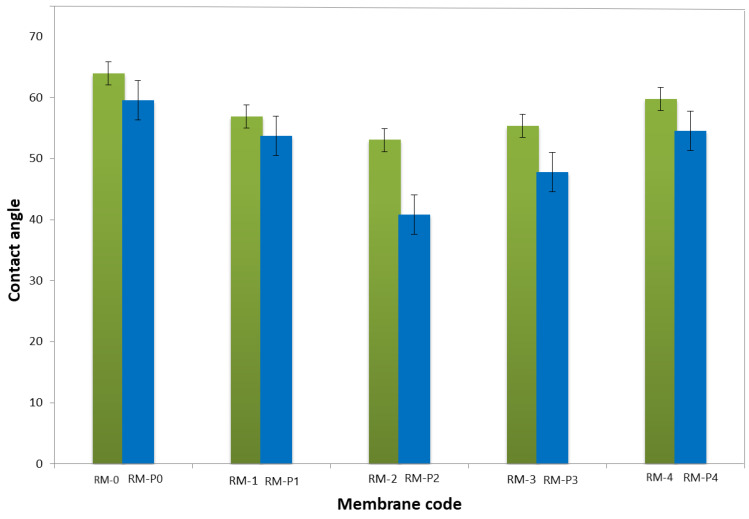
Contact angle of PPSU and PPSU/PVP membrane with various contents of GO-WO_2.89_wt. (%).

The membrane water contact angle (CA) is a great indicator of hydrophilic nature. Low contact angle denotes a high hydrophilic membrane [4]. Figure 9 shows the contact angles (CAs) of PPSU membranes with varying GO-WO_2.89_ nanoparticle concentrations in the polymer casting solution. The decrease in contact angle with a steady increase in the modified GO-WO_2.89_ content can be related to enhancing the hydrophilic property of the surface of the membrane resulting from the hydrophilic property of GO-WO_2.89_. CA decreased from 63° for pure PPSU membrane to 56.9° and 53.06° for 0.05 wt.% and 0.1 wt.% due to absorbing water via the hydrophilic pores of GO via capillary influences. In the other context, increasing the modified GO-WO_2.89_ concentration resulted in an increase in the CA to 55.37° and 59.8° for 0.15 wt.% and 0.2 wt.% of modified GO-WO_2.89_, respectively. At higher concentrations, the contact angle increased because the nanoparticles aggregated and became partially exposed on the membrane surface, which resulted in a blockage of the porous membrane. On the other hand, when PVP was added to the mixture, it was noticed that the CA was lower than without PVP because PVP was added for pore forming, which increased the hydrophilicity of the membrane surface. The CA value decreased from 59.53° for the PPSU/PVP membrane to 53.69° and 40.82° for 0.05 wt.% and 0.1 wt.%. When the concentration of nanoparticles increased, the CA value increased to 47.76° and 54.55° for (0.15 and 0.2) wt.% of nanoparticles, respectively.

**Figure 10:**
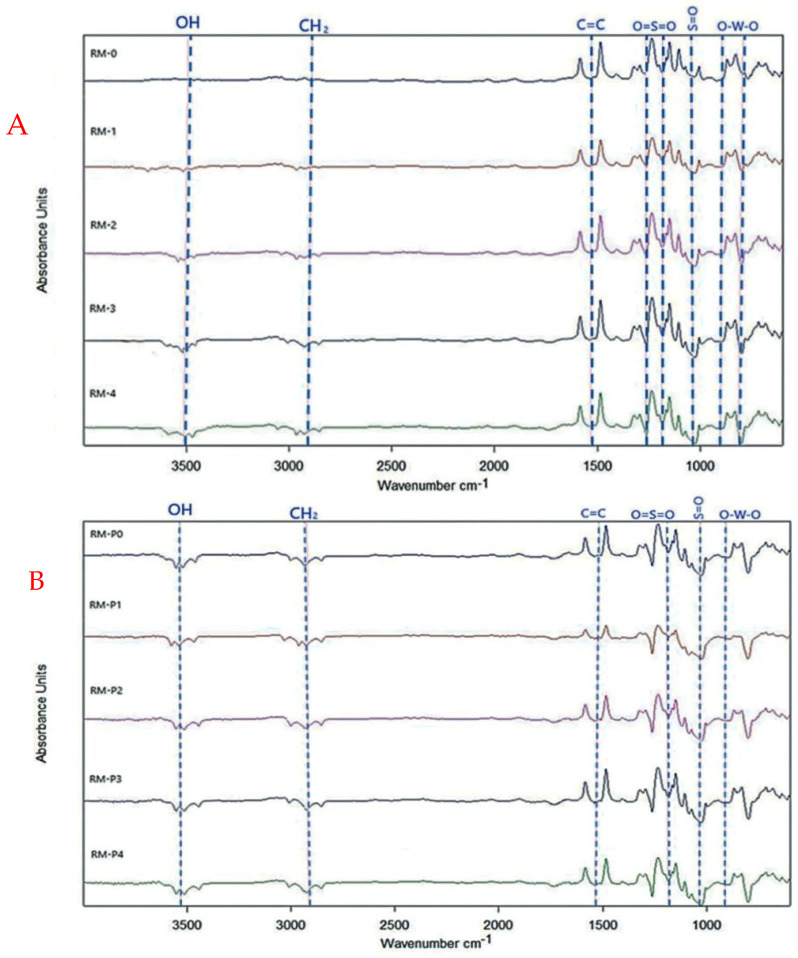
The infrared spectrum of (**A**) PPSU pure and composite membrane, (**B**) PPSU/PVP composite membranes.

FT-IR is one of the many assessment techniques available for identifying the functional groups on a membrane surface and their probable molecular bonds. The impact of GO-WO_2.89_ nanoparticles added to a PPSU polymeric membrane at a percentage of (0–0.2) wt.% and a pure PPSU membrane was investigated. Figure 10A shows the FT-IR spectra of the pure synthetic PPSU and mixed matrix membranes. FT-IR spectra for the membranes were recorded in a range of 4000–400 cm^−1^. The characteristic peaks of infrared asymmetric contraction of the O=S=O functional group were located at 1286 cm^−1^ and 1155 cm^−1^ [43]. At the same time, the S=O groups of PPSU were noticed at around (1106.29–1149.21) cm^−1^. In addition, the peaks at 1486 cm^−1^ and 1585 cm^−1^ are for the C=C stretching vibration of the benzene ring [43]. The OH stretching broad band at 3500 cm^−1^ is related to GO’s hydrophilic character, which could adsorb water droplets via hydrogen bonding. For RM-P0, RM-P1, RM-P2, RM-P3, and RM-P4, Figure 10B shows an absorbance peak at 2950 cm^−1^ being steadily higher due to the CH_2_ for PVP carbonyl absorption and even for 3500 cm^−1^ [62]. The broad peaks observed at 711, 867.23, and 899 cm^−1^ at >1000 cm^−1^ are attributed to O-W-O stretching vibrations of WO_2.89_ [55].

**Figure 11:**
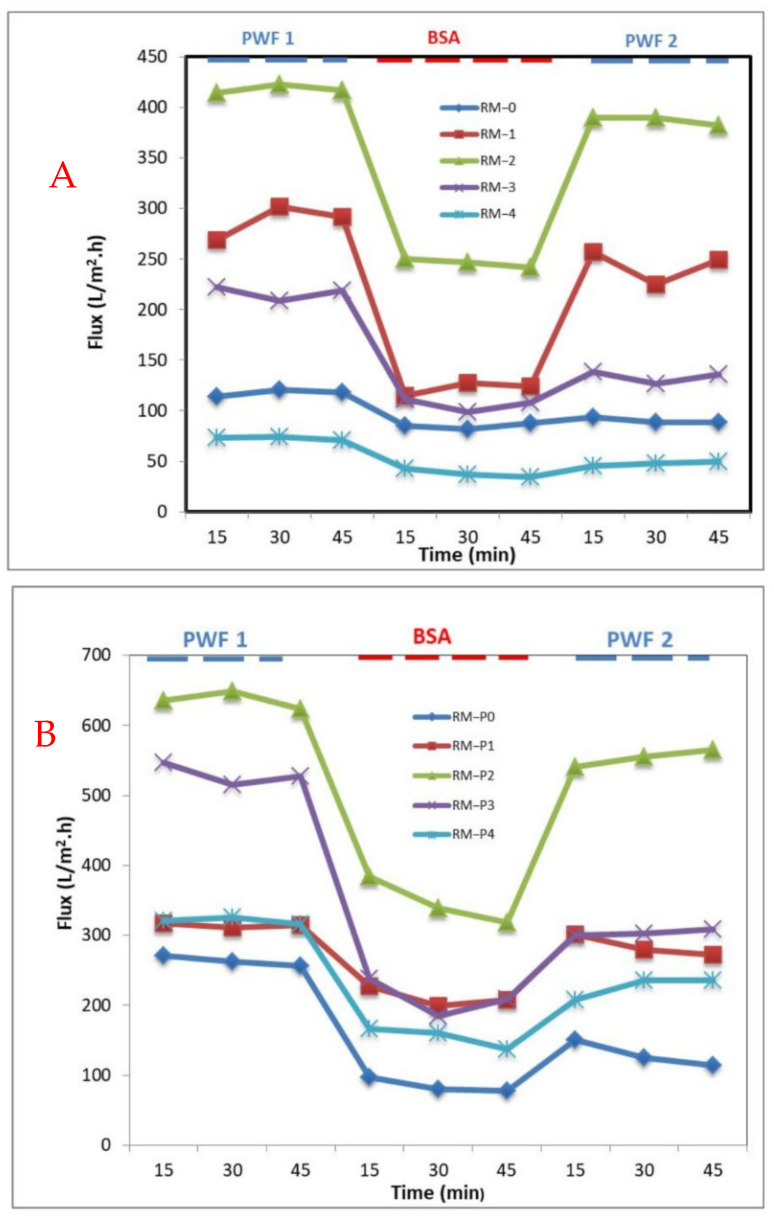
Time−dependent water permeation fluxes during the fouling processes with various wt.% of GO−WO_2.89_ for (**A**) PPSU membrane and (**B**) PPSU/PVP membrane.

The time flux change curve of the PPSU/GO-WO_2.89_ and PPSU/PVP/GO-WO_2.89_ mixed matrix membrane after BSA solution contamination is shown in Figure 11A,B, respectively. When pure water was replaced with BSA solution, the flux of the membrane matrix decreased due to the adsorption of the BSA solution on the surface of the membrane, which led to clogging of the membrane pores. The water flux was restored when the membrane was washed with water, as well as the water flux being stable. The results show that adding GO-WO_2.89_ to the membranes improved their hydrophilic nature, especially for RM-1 and RM-2 for the PPSU membrane and RM-P1 for the PPSU/PVP membrane. As a result, the achievement of antifouling phenomena may be predicted because of an increase in hydrophilic characteristics and the adsorption of fouling particles on the hydrophobic surface. This is because the hydrophilic surface could adsorb water droplets and form a hydro layer, which reduced the adsorption of fouling agents to the membrane’s surface.

**Figure 12:**
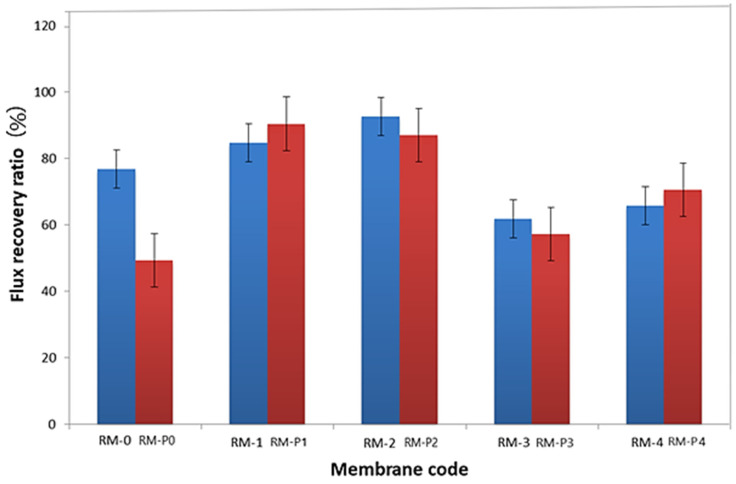
Water flux recovery ratio (FRR %).

The measured reversible fouling resistance (R_r_), irreversible fouling resistance (R_ir_), total fouling resistance (R_t_), and flux recovery ratio (FRR) values are shown in Table 4. The performance of Rir or FRR is commonly used to assess fouling phenomena. As shown in Figure 12, the higher FRR values indicate relatively low permanent protein adsorption to ultrafiltration membranes [63]. It can be noticed that the FRR% increased with the increase in the addition of GO-WO_2.89_ and the higher value was 92.66% at 0.1 wt.% for the PPSU membrane and then decreased with a further increase in the amount of additive. For the PPSU/PVP membrane, the higher value of FRR% was 90.43% and 87.06% at 0.05 wt.% and 0.1 wt.% and then it decreased to 57.29% and 70.50% at 0.15 wt.% and 0.2 wt.% of GO-WO_2.89_, respectively. As a result, the addition of GO-WO_2.89_ improved the membrane’s hydrophilicity, which can improve antifouling performance. It can be indicated that the RM-2 and RM-P2 have the best antifouling properties, which is effective with FRR results for this type of membrane.

**Figure 13:**
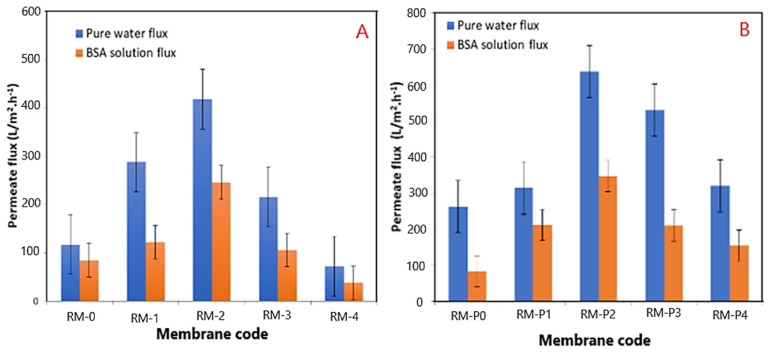
(**A**) Effects of GO−WO_2.89_ loading on PPSU membrane on pure water and BSA solution permeate flux; (**B**) effects of GO−WO_2.89_ loading on PPSU/PVP membrane on pure water and BSA solution permeate flux.

**Figure 14:**
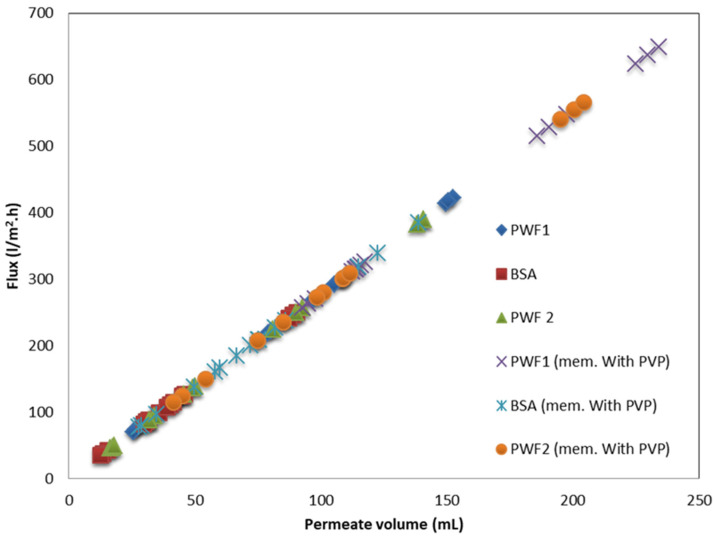
Permeate flux with respect to total permeate volume of the membrane.

The impact of modified GO-WO_2.89_ nanoparticle contents in PPSU solution and PPSU/PVP solution on the pure water permeate flux, BSA solution waste permeates flux, and rejection (R%) of BSA solution wastes at an initial BSA concentration of 1 g/L in water, an applied pressure of 2 bar, a temperature of 25 °C, and an effective membrane area of 14.4 cm^2^, are described in Figure 13A,B. It can be noticed that membranes loaded with GO-WO_2.89_ nanoparticles at 0.05–0.1 wt.% became more hydrophilic and had higher flux than the pristine PPSU membrane. The permeate flux of pure water and BSA solution waste reached 417.96 L·m^−2^·h^−1^ and 246.10 L·m^−2^·h^−1^, respectively. For the PPSU/PVP membrane, the permeate flux reached 636.01 L·m^−2^·h^−1^ and 347.48 L·m^−2^·h^−1^ for pure water and BSA solution waste, respectively, when the GO-WO_2.89_ content reached 0.1 wt.%. The flux for pure water in pristine PPSU membrane was 117.75 L·m^−2^·h^−1^, and it was 85.13 L·m^−2^·h^−1^ for BSA solution waste. PPSU/PVP membrane flux for pure water and BSA solution waste was 263.45 L·m^−2^·h^−1^ and 84.65 L·m^−2^·h^−1^, respectively. The permeate flux increase was due to the membrane’s hydrophilic nature and pore size. After mixing GO-WO_2.89,_ the CA is not the only factor that affects the flux. As can be seen in Table 3 and the cross-section image in Figure 8, the porosity is high enough to allow the solution to pass through the membrane, and the pores become a finger-like structure when the content of the nanoparticles increased to 0.1 wt.% for RM2 as well as for RM-P2 that incorporated with the PVP polymer, responsible for increasing the porosity and the hydrophilic nature of the membrane that will affect the increase in the permeate flux. Furthermore, as shown in Table 3, the thickness of the membrane decreased for the content of 0.05 to 0.1 wt.%. The membrane’s hydrophilicity can explain this increase due to the incorporation of GO-WO_2.89_ nanoparticles and sufficient ion exchange sites. Flow rates across highly hydrophilic membranes are quite high. In other words, loading GO-WO_2.89_ nanoparticles within these ratios strongly affected the surface hydrophilicity and membrane structures, which become two significant variables affecting permeability and selectivity. The increase in the surface hydrophilic nature positively affects water permeation flux [4]. Moreover, increasing the concentration of GO-WO_2.89_ to 0.2 wt.% resulted in a gradual reduction in the permeate flux. The permeate flux was reduced to 216.33 and 72.69 L·m^−2^·h^−1^ for pure water and 106.21 and 38.26 L·m^−2^·h^−1^ for BSA solution for the PPSU membrane. Further, it was 529.93 and 320.76 L·m^−2^·h^−1^ for pure water and 210.68 and 154.98 L·m^−2^·h^−1^ for BSA solution waste for the PPSU/PVP membrane when the GO-WO_2.89_ content reached 0.15 and 0.2 wt.%, respectively. The decline in permeate flow was caused by nanoparticle aggregation on membrane surfaces. The possible explanation for varying concentrations of nanoparticles is to investigate nanoparticle agglomeration in the membranes. Nanoparticle agglomeration is a significant issue that would result in unacceptable changes in the membrane’s properties, such as decreased water flux and mechanical strength. To avoid the problem, many studies recommend a relatively low quantity of nanoparticles [4,63]. Also as notice in Figure 14 there is a relation between the permeation flux and permeate volume so when increasing the volume it will be increase the permeate flux of the membranes, respect to Equation (1). 

**Figure 15:**
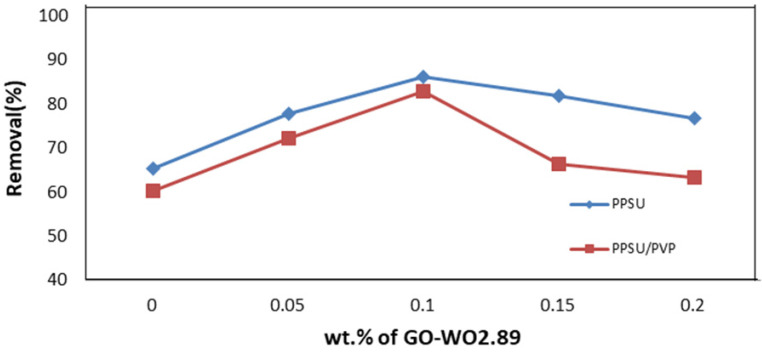
Rejection (R %) for BSA solution by PPSU membrane and PPSU/PVP membrane.

Figure 14 shows the permeation flux is related to the permeate volume linearly. As shown in Figure 15, the rejection percentage (R%) for the pure PPSU membrane initially was 65.28% and 60.1% for the PPSU/PVP membrane but it increased as the loading of GO-WO_2.89_ increased. The R% values for the PPSU membrane increased to 77.699% and 85.97%, and for the PPSU/PVP membrane were 72% and 82.87% at loading amounts of 0.05 and 0.1 wt.%, respectively. The R% decreased slightly at the loading amounts of 0.15 and 0.2 wt.% of GO-WO_2.89_ 81.837% and 76.66% for the PPSU membrane and 66.31% and 63.21% for the PPSU/PVP membrane, respectively. The highest rejection obtained was 85.97% for the PPSU membrane and 82.87% for the PPSU/PVP membrane at a loading value of 0.1 wt.%. The enhanced rejection of BSA was because of an increase in the adsorption sites provided by GO-WO_2.89_ in membrane texture. The aggregation of GO-WO_2.89_ nanoparticles on the top layer of the membranes at 0.15 and 0.2 wt.% of NPs was responsible for the decrease in the rejection R% of BSA. Li guang et al. [64] reported a decrease in the rejection of BSA solution after the agglomeration of nanoparticles on the polymeric membrane.

Table 5 compares the membrane performance of PPSU modified with GO-WO_2.89_ that was presented in the current research with another study that used BSA solution as a removal to test the performance of the membrane. Also shown in Table 5 are the main characteristics of the nanocomposite membranes, including contact angle, flux, and porosity. Comparing the PPSU/GO-WO_2.89_ membranes to another study, they have such a perfectly rational solution permeation flux. Moreover, Table 5 shows that the contact angle for GO-WO_2.89_ in the current study was 53.06° for RM-2 and 40.82° for RM-P2, whereas the contact angle for the GO in the casting solution of 1.5 wt.% in the PPSU membrane reported was 67.1° by Xiao et al. [43]. The information presented here leads to the conclusion that the quantity of nanoparticles should be optimized in order to achieve the highest membrane performance values.

## 4. Conclusions

The current study includes the preparation of novel GO-WO_2.89_ nanoparticles to modify PPSU and PPSU/PVP ultrafiltration membranes. A thorough characterization was employed to analyze the membrane’s performance against BSA solution as a fouling solution. The SEM images show that the PPSU and PPSU/PVP membrane structure significantly changes with the addition of GO-WO_2.89_ at a concentration of 0.05 and 0.1 wt.% in the casting solution. The hydrophilicity and porosity of the membrane were highly improved as the concentration of GO-WO_2.89_ nanoparticles increased in the PPSU casting solution to 0.05 and 0.1 wt.%. The prepared membranes of 0.1 wt.%, with and without water-soluble PVP, were the most efficient in terms of performance and antifouling ability. The RM-2 and RM-P2 membranes had the maximum flux of pure water and BSA rejection at 0.1 wt.% of 417.96 and 636.01 L·m^−2^·h^−1^ and 85.97% and 82.87, respectively. Furthermore, self-cleaning characteristics and the antifouling performance of the RM-2 and RM-P2 membranes were excellent, with a flux recovery ratio of up to 92.66% and 87.06%, respectively. In addition, the WO_2.89_ added an incredible antibacterial effect to GO so it can be used to treat biologically contaminated wastewater.

## Figures and Tables

**Table 1:** Composition of polymer, additives, and solvent for membrane fabrication.

Membrane Code	PPSU%	PVP%	GO-WO_2.89_%	DMAC Solution%
RM-0	17	0	0	83
RM-1	17	0	0.05	82.95
RM-2	17	0	0.1	82.9
RM-3	17	0	0.15	82.85
RM-4	17	0	0.2	82.8
RM-P0	15	2	0	83
RM-P1	15	2	0.05	82.95
RM-P2	15	2	0.1	82.9
RM-P3	15	2	0.15	82.85
RM-P4	15	2	0.2	82.8

**Table 2:** Number and percent of antibacterial effect by GO-WO_2.89_ nanoparticles.

Conc.	NOB	Percent of Anti-Bacteria
2 mg	8	92%
4 mg	9	91%
6 mg	10	90%

**Table 3:** The thickness and the porosity of the membrane.

Membrane Code	Thickness (cm)	Porosity (%)
RM-0	0.01247	33.4
RM-1	0.01007	66.1
RM-2	0.00975	81.4
RM-3	0.01526	79.2
RM-4	0.01043	44.2
RM-P0	0.01363	84.9
RM-P1	0.01219	90.7
RM-P2	0.01048	92.9
RM-P3	0.01639	91.9
RM-P4	0.00949	90.1

**Table 4:** Antifouling properties of prepared membranes.

Membrane	J_w1_ (L /m^2^h)	J_p_ (L /m^2^h)	J_w2_ (L /m^2^h)	R_r_	R_ir_	R_t_	FRR%
RM-0	117.7	85.1	90.3	4.4	23.3	27.7	76.7
RM-1	287.7	122.2	243.6	42.2	15.3	57.5	84.7
RM-2	417.9	246.1	387.3	33.8	7.3	41.1	92.7
RM-3	216.3	106.2	133.8	12.8	38.1	50.9	61.9
RM-4	72.7	38.3	47.8	13.1	34.2	47.4	65.7
RM-P0	263.5	84.7	129.8	17.1	50.7	67.9	49.3
RM-P1	314.8	211.8	284.6	23.2	9.6	32.7	90.4
RM-P2	636.0	347.4	553.8	32.4	12.9	45.4	87.1
RM-P3	529.9	210.7	303.6	17.5	42.7	60.2	57.3
RM-P4	320.8	154.9	226.2	22.2	29.5	51.7	70.5

**Table 5:** Demonstrates the comparison of this work with recently published work.

Type of Polymer	Type and Composition of NPs	%Porosity	Contact Angle	Flux (L/m^2^·h)	%Rejection	Ref.
PVC	3 wt.% ZnO	79.8%	54.5°	401.9 kg/m^2^·h	97.5% BSA	[65]
PES	ZrO_2_ (1%wt)		52.3°	83.6 L/m^2^·h	92.7% BSA 91.2% OVA	[66]
PES	TiO_2_/F127	91.3%	61.2°	235.9 L/m^2^·h	96% BSA	[67]
PES	CuO/ZnO (0.2%)		65.5°	679 kg/m^2^·h	99% BSA	[68]
PES	CuO (0.1%wt)		64°	869.9 kg/m^2^·h	97% BSA	[69]
PVDF	TiO2 (<2 wt.%)		76°	111.7 L/m^2^·h	100% BSA	[70]
EPVC/PEG	TiO2 (2 wt.%)	78.7%	57.2°	435 kg/m^2^·h	98% BSA	[71]
PVDF	GO-PVP		68°	104.3 L/m^2^·h	85% BSA	[72]
PPSU	1.5 wt.% GO	63.7%	67.1°	231.7 L/m^2^·h	95% BAS	[43]
PPSU	0.1 wt.% GO-WO_2.89_	81.4%	53.06°	246.1 L/m^2^·h	85.9% BSA	This paper
PPSU	0.1 wt.% PVP/GO-WO_2.89_	92.9%	40.82°	347.4 L/m^2^·h	82.8%BSA	This paper

## Data Availability

Not applicable.
